# Genome Sequence of Enterovirus D68 and Clinical Disease, Thailand

**DOI:** 10.3201/eid2102.141742

**Published:** 2015-02

**Authors:** Sompong Vongpunsawad, Slinporn Prachayangprecha, Jira Chansaenroj, Bart L. Haagmans, Saskia L. Smits, Yong Poovorawan

**Affiliations:** Chulalongkorn University, Bangkok, Thailand (S. Vongpunsawad, S. Prachayangprecha, J. Chansaenroj, Y. Poovorawan);; Erasmus Medical Center, Rotterdam, the Netherlands (B.L. Haagmans, S.L. Smits)

**Keywords:** enterovirus D68, enterovirus, viruses, respiratory illness, genome sequence, St. Louis, Missouri, Thailand

**To the Editor:** Outbreaks of respiratory enterovirus D68 infection were particularly severe in 2014 in the United States. Wylie et al. recently analyzed the whole genomes of clinical strains from St. Louis, Missouri, USA, and the US Centers for Disease Control and Prevention (Atlanta, GA, USA) (*1*). Results showed that the most closely related genomes to the St. Louis strains were strains CU134 (GenBank accession no. KM361523) and CU171 (KM361524), which were identified in Thailand in 2011 (*2*,*3*).

To provide additional background regarding the origin of these strains from Thailand, including 1 additional CU70 strain (KM361525), we report clinical features of the 3 patients from which the strains were derived. This additional information might assist clinical scientists in early recognition of enterovirus D68 infections and provide insight into viral pathogenesis.

The 3 patients (1 boy and 2 girls; age range 7–24 months) were hospitalized during July–September 2011 with pneumonia. At admission, they had cough, rhinorrhea, and dyspnea. Fever, crepitation, and wheezing were observed in patients CU70 and CU134. Patients CU134 and CU171 had suprasternal and subcostal retraction, and patient CU171 had signs of nasal flaring and inspiratory stridor (he has an underlying double aortic arch). Chest radiographs showed perihilar infiltration for patients CU70 and CU134. Hemocultures and test results for respiratory viruses for all 3 patients were negative (*2*).

Physicians provided respiratory support to all 3 patients by oxygen flow and nebulized bronchodilator. In addition, patient CU171 was given nebulized adrenaline, an intravenous corticosteroid, and intravenous antimicrobial drugs. Patients CU70 and CU134 were discharged after 3 and 8 days, respectively. However, patient CU171 remained hospitalized for 16 days.

Nasopharyngeal aspirates obtained from the 3 patients were subjected to next-generation sequencing and genomic analysis. From the total number of analyzed reads for isolates from patients CU70 (n = 10,482), CU134 (n = 11,504), and CU171 (n = 4,545), ≈1,100–1,600 enterovirus D68 sequence reads were identified. Anellovirus sequences (n < 60) were found in aspirates from patients CU70 and CU171. Furthermore, aspirates from patients CU134 and CU171 contained human rhinovirus B (n = 73) and human rhinovirus C (n = 15), respectively (*2*). Future genomic studies and surveillance of enterovirus D68 will be helpful in monitoring its spread next season.
